# Modification of the Emergency Severity Index Improves Mortality Prediction in Older Patients

**DOI:** 10.5811/westjem.2019.4.40031

**Published:** 2019-07-02

**Authors:** Alexandra Malinovska, Laurentia Pitasch, Nicolas Geigy, Christian H. Nickel, Roland Bingisser

**Affiliations:** * University Hospital Basel, Department of Emergency Medicine, Basel, Switzerland; † Liestal Cantonal Hospital, Department of Emergency Medicine, Liestal, Switzerland

## Abstract

**Introduction:**

Older patients frequently present to the emergency department (ED) with nonspecific complaints (NSC), such as generalized weakness. They are at risk of adverse outcomes, and early risk stratification is crucial. Triage using Emergency Severity Index (ESI) is reliable and valid, but older patients are prone to undertriage, most often at decision point D. The aim of this study was to assess the predictive power of additional clinical parameters in NSC patients.

**Methods:**

Baseline demographics, vital signs, and deterioration of activity of daily living (ADL) in patients with NSC were prospectively assessed at four EDs. Physicians scored the coherence of history and their first impression. For prediction of 30-day mortality, we combined vital signs at decision point D (heart rate, respiratory rate, oxygen saturation) as “ESI vital,” and added “ADL deterioration,” “incoherence of history,” or “first impression,” using logistic regression models.

**Results:**

We included 948 patients with a median age of 81 years, 62% of whom were female. The baseline parameters at decision point D (ESI vital) showed an area under the curve (AUC) of 0.64 for predicting 30-day mortality in NSC patients. AUCs increased to 0.67 by adding ADL deterioration to 0.66 by adding incoherence of history, and to 0.71 by adding first impression. Maximal AUC was 0.73, combining all parameters.

**Conclusion:**

Adding the physicians’ first impressions to vital signs at decision point D increases predictive power of 30-day mortality significantly. Therefore, a modified ESI could improve predictive power of triage in older patients presenting with NSCs.

## INTRODUCTION

Over 15% of the visits to emergency departments (EDs) can be attributed to patients older than 65 years in the United States.^1^ Compared to younger patients, this population has a higher risk of death or development of a functional decline leading to institutionalization.^2^,^3^ Moreover, atypical symptoms in prevalent diseases are common, and older patients often present with nonspecific complaints (NSC), such as weakness or acute functional decline.^4^,^5^ Previous studies showed that patients presenting with NSCs have a 30-day mortality reaching 6%–13%.^6^,^7^ However, individual prediction of mortality is difficult due to the broad differential diagnosis including a wide span of reasons ranging from lack of social support to acute life-threatening disease.^4^,^8^ Therefore, disease-specific risk scores (such as the HEART score) are not used at presentation; instead, general risk stratification tools to identify patients at risk should be developed. Although various parameters and clinical tools for the prediction of mortality in the general ED population exist,^9^ risk stratification tools for older patients with NCSs have not yet been developed. This is an unmet need in one of the largest groups that requires several external resources during work-up presenting to the ED.^10^

The main tool used for the prediction of mortality in ED “all-comers” population is triage.^11^ Triage is the categorization of patients according to urgency and prognosis at presentation. A reliable and valid triage instrument is the Emergency Severity Index (ESI), which uses a five-level classification system.^11^,^12^ ESI levels can be used to predict six-month and one-year mortality in an older ED population.^13^,^14^ Four decision points (A to D) are used to triage patients into the five ESI levels. Patients with ESI level 1 are in need of an immediate life-saving intervention (decision point A). Stable patients in a high-risk situation are designated to ESI level 2 (decision point B). At decision point C, patients are assigned according to the expected use of resources, reaching from none (ESI 5) to more than one (ESI 3). To ultimately classify a patient as ESI level 3, vital signs must be assessed. If they exceed defined limits, re-assignment to ESI level 2 is to be considered (decision point D).^11^,^15^ Obviously, vital sign assessment is important for the identification of patients with a poorer prognosis requiring urgent attention.

One of the main problems of triage in older patients is undertriage.^10^ Undertriage describes a phenomenon where patients are misclassified into a lower urgency group. It occurs most commonly at decision point D separating ESI 3 from ESI 2.^10^ This highlights that decision point D may be crucial to avoiding undertriage and could, therefore, be a weakness of this triage tool.

The aim of this study was to assess the effectiveness of vital sign assessments at decision point D (“ESI vital”) for the prediction of 30-day mortality in patients presenting with NSCs. We further evaluated the predictive power at decision point D with additional parameters such as the deterioration in activity of daily living (ADL) (“ESI A”), the incoherence of history (“ESI H”), and the first impression by the physician (“ESI F”). We focused on older patients with NSCs as a highly prevalent and vulnerable population.

## METHODS

### Study Design and Setting

This study was part of a prospective, observational multicenter study with a 30-day follow-up. Data collection was conducted from May 24, 2007 to July 26, 2011. The study was performed at three EDs: a 700-bed tertiary care hospital, a 600-bed tertiary care hospital, and a 400-bed secondary care hospital. The local ethics committee approved the study protocol. The study is registered at ClinicalTrials.gov.

### Selection of Participants

We used a validated German version of the Emergency Severity Index (ESI) for triage.^11^,^16^ All non-trauma patients older than 18 years with an ESI of 2 or 3, whose vital signs were not extremely out of range, (see Table 1) and who presented to the ED with NSCs were eligible for this study. Patients were included by the study team after recording of the patient’s medical history and focused clinical examination, but before laboratory results were available. Exclusion criteria are shown in Table 1.

**Population Health Research Capsule**

What do we already know about this issue?

Patients presenting with nonspecific complaints (NSCs) in the emergency department (ED) are older than average and show an increased risk of adverse outcomes.

What was the research question?

The aim was to assess the effectiveness of parameters for the prediction of 30-day mortality in NSCs patients.

What was the major finding of the study?

The parameters of respiratory rate and the physician’s “gesalt” are most efficient in predicting 30-day mortality.

How does this improve population health?

The parameters can help improve the effectiveness of triaging patients according to their risk and assign the appropriate resources for each patient.

### Screening for Nonspecific Complaints (NSCs)

NSCs are symptoms that are not part of the set of specific complaints. Patients with specific complaints or a clinical presentation suggestive of a working diagnosis can be managed using evidence-based management protocols for emergency physicians. Patients for whom the physicians could name a specific complaint or a specific working diagnosis were excluded from the study. Any patients presenting with recent external laboratory results or specific electrocardiogram (ECG) changes on admission were not eligible.^4^ This definition for NSCs by exclusion has a major advantage, as there is not an endless list of nonspecific presenting complaints. Furthermore, patients with NSCs as defined above are comparable to patients with weakness and fatigue regarding demographics and outcomes.^17^

### Measurements

Previously trained study physicians recorded the following data shortly after ED presentation: demographic (age, sex); ESI level; vital signs (heart rate, blood pressure, oxygen saturation, and body temperature); ECG; ADL deterioration within the prior two weeks; evaluation of the coherence of the patient’s history; and the physician’s first impression of the patient. The mode of presentation was extracted from the patient’s electronic health records (EHR) and included two modes: “ambulance transport” (including hospital transfer) or “walk-in.”

To assess the parameter “ADL deterioration,” the study physician asked the patient about a decline of independence in the prior two weeks regarding “bathing,” “dressing,” “mobility,” “feeding,” “toilet hygiene,” and “incontinence.” For the parameter “coherence of history,” the study physicians provided a subjective judgment (yes/no), whether they considered the history given by the patient as coherent (no discrepancy to other information, such as health records or histories by proxies). For “first impression by the physician,” every physician assigned points to the question, “how ill does this patient look?,” using a scale ranging from 0 (patient looks very healthy) to 10 (patient looks critically ill). Mortality at 30 days was obtained from the EHR, the patient’s primary care physicians (by questionnaires), and from hospital discharge reports.

### Emergency Severity Index

Specifically trained triage nurses used the German version of the ESI.^11^ The first of the four decision points (decision point A) distinguishes patients in need of an immediate life-saving intervention and allocates them to ESI level 1. ESI level 2 is assigned to patients who should not wait due to high-risk situations, such as new onset of confusion, lethargy, disorientation, and severe pain or distress (decision point B). ESI levels 5 to 3 are assigned according to the expected use of resources. Patients who need no resources are categorized as level ESI 5, while those who need one or more resources are classified as ESI 4 or ESI 3, respectively (decision point C). If patients are to be assigned to ESI level 3, vital signs must be assessed. If vital signs exceed the defined limits (heart rate higher than 100 beats per minute (min), respiratory rate higher than 20/min, or oxygen saturation lower than 92%), re-assignment to ESI level 2 should be considered (decision point D).^11^,^15^

### Additional Parameters Assessed at Decision Point D

For prediction of outcome, we compared the following predictors: all parameters in the set of vital signs at decision point D (heart rate, respiratory rate, and oxygen saturation [“ESI vital”]). Additional possible outcome predictors were a decline in ADL (“ESI A”), an incoherent medical history (“ESI H”), and the first impression by the physician of 9 or higher (“ESI F”). Moreover, we added the additional parameters pairwise to obtain “ESI AH,” “ESI AF,” and “ESI HF,” as well as all additional parameters combined to obtain “ESI AHF” as an outcome predictor.

### Statistical Analyses

We tested the following 12 parameters to predict 30-day mortality in the NSCs population: age (years); sex (male); heart rate (per min); respiratory rate (per min); oxygen saturation (% on room air); systolic blood pressure (mmHg); low temperature (<35°C); ECG changes (all findings except tachycardia, bradycardia, and pacemaker rhythm); “ambulance transport” mode of presentation; “incoherence of history”; “ADL deterioration”; and “first impression by the physician.” In order to detect the effect of various parameters on the 30-day mortality, we used univariate logistic regression models. Results are expressed as odds ratios (OR) with corresponding 95% confidence intervals (CI) and p-values. Further, the AUC of a receiver operating characteristic (ROC) curve was calculated with corresponding 95% CIs for each parameter.

To compare the different scores (eg, “ESI vital,” “ESI A,” etc.), AUCs of the different scores were calculated and compared pairwise using a non-parametric approach model.^18^ We computed descriptive statistics with frequencies or median and interquartile range (IQR). Overall p-values correspond to t-test (for means), Kruskall-Wallis test (for median), and chi-square test or Fisher’s exact test if the expected frequencies were less than 5. A p-value <0.05 was considered significant. We did all analyses using R version 3.0.1 (The R Foundation for Statistical Computing, Vienna, Austria).

## RESULTS

### Characteristics of Study Subjects

**Figure 1:**
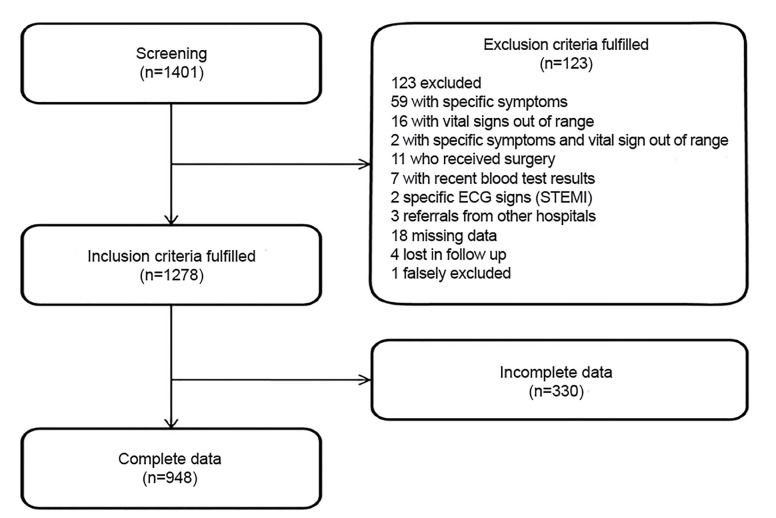
Patient enrollment chart. *ECG*, electrocardiogram; *STEMI*; ST-elevation myocardial infarction.

A total of 1401 non-trauma patients who presented with NSCs to the ED were screened for eligibility. Of these, 1278 patients fulfilled the inclusion criteria. The data were retrospectively reviewed for completeness of all clinical parameters. In 330 patients one or more clinical parameters was missing, and these patients were subsequently excluded (Figure 1). To exclude selection bias, we compared the 948 patients with complete data and the 330 patients with incomplete data. The two groups were comparable with respect to age, sex, and vital signs (heart rate, respiratory rate, oxygen saturation, and systolic blood pressure), and regarding the occurrence of the parameters “ADL deterioration,” “incoherence of history,” “first impression by the physician,” and 30-day mortality (Supplementary Data 1).

Table 2 shows the baseline characteristics of all 948 patients included. A total of 589 patients (62.1%) were female; the median age was 81 years with an IQR from 74–87 years, and 835 (88.1%) patients were older than 65 years. A total of 57 (6.01%) patients were not alive 30 days after presentation to the ED.

### Prediction of Mortality

To determine predictors of mortality, we analyzed the effectiveness of 12 clinical parameters including the vital sign parameters assessed at decision point D. We performed univariate logistic regression analysis for all parameters (Table 3). We found the following parameters to predict mortality: sex (male); respiratory rate; “ADL deterioration”; “incoherence of history”; and “first impression by the physician >8 points.” Of these, the physician’s first impression had the best predictive performance regarding 30-day mortality with an OR of 1.250 per 10% increase and an AUC of 0.67. The second most reliable parameter was respiratory rate with an OR of 2.667 and an AUC of 0.56. Respiratory rate is one of the vital signs assessed at decision point D. The other two vital signs recorded at decision point D (ie, heart rate and oxygen saturation had low predictive power) (Table 3).

To test the predictive power of vital signs at decision point D, we combined all three vital signs (respiratory rate, heart rate, and oxygen saturation) and calculated the “ESI vital” score. This score yielded an AUC of 0.64 for predicting the 30-day mortality in patients with NSCs.

**Figure 2:**
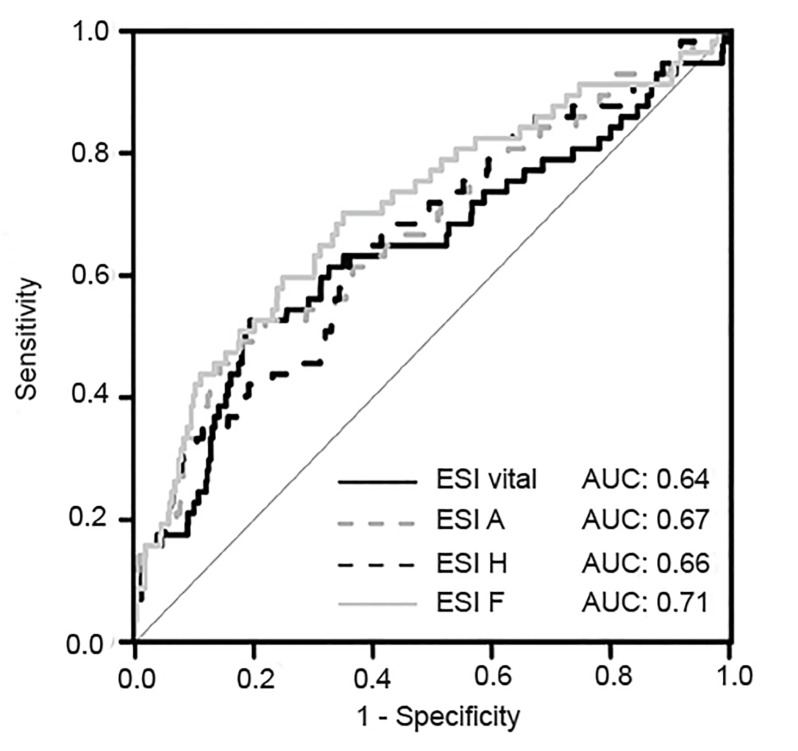
Comparison of area under the receiver operating characteristic (ROC) curve of the different modifications of the emergency severity index (ESI) vital, ESI A, ESI H, and ESI F. The black continuous line shows the ESI vital including pulse, respiratory rate, and oxygen-saturation. The grey dotted line shows the ESI A including ESI vital and activity of daily living deterioration. The black dotted line shows ESI H including ESI vital and accuracy of medical history. The grey continuous line shows ESI F including ESI vital and the first impression by the physician. *AUC*, area under the curve; *ESI A*, decline in activity of daily living (ADL); *ESI H*, incoherence of history; *ESI F*, first impression of physician.

To further increase the predictive power of “ESI vital,” we added the three remaining best-performing parameters (accessory parameters) to the score: “ADL deterioration” for “ESI A”; “incoherence of history” for “ESI H”; and “first impression by the physician >8 points” for “ESI F.” We calculated the predictive power of the different scores and compared it to the power of “ESI vital.” The AUC increased from 0.64 to 0.67 for “ESI A” and to 0.66 for “ESI H.” A significant increase to 0.71 was observed for “ESI F” (p=0.004), if >8 points was chosen as cut-off. Figure 2 shows the ROC curves for 30-day mortality based on the scores “ESI vital,” “ESI A,” “ESI H,” and “ESI F.” This shows that the prediction of the 30-day mortality could be increased by adding the parameters “ADL deterioration,” “incoherence of history,” or “first impression by the physician” to the basic model “ESI vital.”

To further increase the predictive power, we added the additional parameters pairwise to “ESI vital”: “ADL deterioration” and “incoherence of history” for “ESI AH;” “incoherence of history” and “first impression by the physician” for “ESI HF”; and “ADL deterioration” and “first impression by the physician” for “ESI AF.” We compared the AUC of these scores with the AUCs of “ESI vital” and observed an increase in the predictive power, whereby “ESI AH” had an AUC of 0.68, “ESI HF” of 0.72, and “ESI AF” of 0.72. Moreover, we added all three parameters to “ESI vital” (“ESI AHF”). By combining all three parameters, the predictive power further increased to an AUC of 0.73. This shows that the predictive power of “ESI vital” can be increased by addition of multiple accessory parameters, whereby the combination “ESI AHF” performed best, but not significantly better than ESI F.

## DISCUSSION

We analyzed the parameters determined in ESI triage at decision point D separately and found that first, respiratory rate alone can efficiently predict 30-day mortality in patients presenting with NSCs. This result is in line with previous studies, showing that abnormal respiratory rate (<8/min and >30/min) can be used to predict in-hospital mortality.^19^,^20^ Tachycardia and hypoxia, the other two parameters assessed at decision point D, were found previously to be associated with an increased risk of death in the ED.^20^ However, these two parameters alone were not useful for risk prediction in our population of elderly patients with NSCs. This discrepancy can be attributed to the a priori exclusion of patients with severe tachycardia and hypoxia (Table 1).

Second, the combination of all three vital sign parameters measured at decision point D (heart rate, respiratory rate, and oxygenation), predicted 30-day mortality moderately well. This result is also in line with previous studies.^21^,^22^ Yet undertriage occurs most often at decision point D.^10^ The reasons for this are probably multifactorial. However, it appears that vital sign assessment at decision point D is often performed incompletely, and this lack of adherence to the algorithm (e.g., lack of measurement of respiratory rate) contributes to the occurrence of undertriage.^22^ Hence, we have shown that vital signs are predictive for the patients’ outcomes, and our findings support the importance of a complete assessment of vital signs, including respiratory rate, to avoid undertriage.

Older patients are particularly at risk of undertriage.^10^ This might be explained by the age-related changes of vital signs in the elderly.^23^ Moreover, the measurement of vital signs tends to be less sensitive in patients with severe illness or injury who are older than 75 years. Thus, the use of age-adapted vital sign cut-offs at decision point D for the geriatric patient population has been suggested.^24^ Adapting vital sign cut-offs for specific age groups has been applied in the pediatric version of the ESI (e.g., heart rate > 140 [3–8 years], respiratory rate > 30 [3–8 years]).^25^ Adapting cut-offs for vital signs in older patients may increase the power of mortality prediction, but has not been used for any triage tool as of yet.

Third, we could show that a further improvement of mortality prediction can be achieved by adding parameters to the baseline model of vital signs at ESI decision point D. The best-performing additional parameters were “ADL deterioration” and “first impression by the physician.” Consistent with this, adding these parameters to “ESI vital” increased the predictive power of the tool. The increased predictive power of “first impression by the physician” is consistent with the findings of a recent study, which showed that physicians may identify patients at risk of in-hospital mortality.^26^ Adding the parameter “ADL deterioration” also increased the prediction power over the baseline model of vital signs. This is in line with a previous study showing that the decrease of the ADL is a risk factor for in-hospital death in older patients.^20^

The predictor analysis also showed an increased (yet not significant) risk of mortality for patients with an incoherent history (OR 1.53). This is in agreement with other studies which showed that an inaccurate medical history might lead to delayed or missed diagnoses.^27^,^28^

The combination of all three parameters improved the predictive power of “ESI vital.” However, the improvement was comparable to adding “first impression by the physician” only. Additionally adding so many variables into the ESI while testing may limit the external validity. Therefore, we believe that “ESI F” should be preferred over other modifications as it performs best while keeping triage using the ESI as fast and simple as possible.

## LIMITATIONS

Various parameters were needed for the calculation of the different scores. Since data were not available for all patients, 334 patients could not be included to the analysis. This, however, increases the risk of a selection bias. Nonetheless, both included and excluded populations are comparable regarding baseline demographics and regarding the prevalence of the parameters. Therefore, we assume that lack of data had most likely a random effect. Yet excluding patients with vital parameters extremely out of range in the first place could limit the performance of the different scores. Moreover, other tools such as the PARIS score, which is based on blood pressure, age, respiratory rate, loss of independence and oxygen saturation^29^ were not evaluated but could be tested in future studies.

Generally, mortality may not be the ideal outcome parameter in a population of patients with a median age of 82 years. In future studies, the performance of scores should be tested on other outcomes such as acute morbidity or institutionalization.

## CONCLUSION

Patients with NSCs may need a triage system using additional information. At triage, it is important to use easily available parameters requiring no further equipment. We have shown that adding the first impression by the physician increased the prediction of mortality in patients presenting with NSC. Thus, in addition to vital signs out of the defined range, a score of 9 or 10 for “looking ill” may be considered for re-assignment to ESI level 2. With this modification, the use of “gestalt,” which was suggested in the original ESI score, could be specified concisely at decision point D. Improving the predictive power of triage in elderly patients presenting with nonspecific complaints is of importance due to their high vulnerability. This additional specification of decision point D should be prospectively validated in patients older than 65 years.

## Supplementary Information



## Figures and Tables

**Table 1:** Exclusion criteria in study assessing the predictive power of triage in older patients presenting with non-specific complaints.

Criteria	Examples
ESI 1, 4, or 5	
Specific complaints	Chest pain, dyspnea, abdominal pain
Clinical presentation suggestive of a working diagnosis to be managed by evidence based protocols	Jaundice
Vital signs markedly out of range	Systolic blood pressure < 90mmHg Heart rate > 120 beats/min Tympanic body temperature > 38.4°C or <35.6°C Respiratory rate > 30 breaths/min Oxygen saturation < 92%
Recent external laboratory results, or referral	Documented anemia
Specific electrocardiogram changes on ED presentation	ST-segment elevation
Moribund patients with terminal conditions	End-stage cancer
Incomplete data	Missing values for activity of daily living (ADL)
Lack of informed consent	

*ESI*; emergency severity index; *mmHg*, millimeters of mercury; *°C*, degrees Celcius; *min*, minute.

**Table 2:** Baseline and demographic variables.

Variables	Number
Total [n]	948
Sex	
Male [n (%)]	359 (37.9%)
Female [n (%)]	589 (62.1%)
Age [median years (IQR)]	81 (74–87)
< 65 years [n (%)]	113 (11.9%)
≥ 65 years [n (%)]	835 (88.1%)
ESI Level at Triage	
2	41 (4.7%)
3	836 (95.3%)
Mortality (30 days)	
Non-survivors [n (%)]	57 (6.0%)
Living situation	
Nursing home [n (%)]	81 (8.5%)
Vital signs at triage	
Heart rate > 100/min [n (%)]	104 (11.0%)
Respiratory rate > 20/min / < 8/min [n (%)]	93 (9.8%)
O_2_ saturation < 92% [n (%)]	23 (2.4%)
Systolic blood pressure < 100 mmHg [n (%)]	38 (4.0%)
Other parameters at triage [n (%)]	
Incoherence of history [n (%)]	436 (46.0%)
ADL deterioration [n (%)]	544 (57.4%)
First impression > 8 points [n (%)]	75 (7.9%)
Temperature < 36.5°C [n (%)]	6 (0.6%)
ECG changes [n (%)]	447 (47.2%)
Ambulance transport [n (%)]	615 (64.9%)

*O_2_*, oxygen; *mmHg*, millimeters of mercury; *IQR*, interquartile range; *°C*, degrees Celcius; *min*, minute; *ESI*, emergency severity index.

Incoherence of history: physician’s judgment, whether he or she considered the patient’s history as coherent. Activity of daily living (ADL) deterioration: deterioration of any ADL within the prior two weeks. First impression: rating by the physician using a scale ranging from 0 (patient looks very healthy) to 10 (patient looks critically ill). Electrocardiogram (ECG) changes: all findings except tachycardia, bradycardia, and pacemaker rhythm and specific changes.

**Table 3:** Odds ratios (OR) for all parameters tested for an association with 30-day mortality.

Parameters	AUC	Lower	Upper
Age (years)	0.54	0.47	0.62
Sex (male)	0.63	0.57	0.70
Vital signs at triage			
Heart rate > 100/minute	0.57	0.48	0.66
Systolic blood pressure < 100 mmHg	0.61	0.54	0.68
Respiratory rate > 20/minute	0.56	0.51	0.61
O_2_ saturation > 92%	0.50	0.42	0.57
Other parameters at triage			
Incoherence of history	0.55	0.49	0.62
ADL deterioration	0.59	0.53	0.65
First impression > 8 points	0.67	0.60	0.74
Temperature < 36.5°C	0.56	0.48	0.65
ECG changes	0.55	0.48	0.61
Ambulance transport	0.50	0.44	0.56
Scores			
“ESI vital”	0.64	0.56	0.73
“ESI A”	0.67	0.59	0.75
“ESI H”	0.66	0.58	0.74
“ESI F”	0.71	0.63	0.79
“ESI AH”	0.68	0.60	0.76
“ESI HF”	0.72	0.65	0.79
“ESI AF”	0.72	0.64	0.79
“ESI AHF”	0.73	0.65	0.80

*mmHg*, millimeters of mercury; *O_2_*, oxygen; *°C*, degrees Celcius; *ESI*, Emergency Severity Index; *ESI A,* decline in activity of daily living (ADL); *ESI H*, incoherence of history; *ESI F*, first impression of physician; *ESI AH*, decline in ADL plus incoherence of history; *ESI AF*, decline in ADL plus first impression of physician; *ESI HF,* incoherence of history plus first impression of physician; *ESI AHF*, decline in ADL plus incoherence of history plus first impression of physician.

Incoherence of history: physician’s judgment, whether he or she considered the patient’s history as coherent. Activity of daily living (ADL) deterioration: deterioration of any ADL within the prior two weeks. First impression: rating by the physician using a scale ranging from 0 (patient looks very healthy) to 10 (patient looks critically ill). Electrocardiogram (ECG) changes: all findings except tachycardia, bradycardia, and pacemaker rhythm and specific changes.
