# Supplementary material for: Modification of the Emergency Severity Index Improves Mortality Prediction in Older Patients
Source: West J Emerg Med. 2019 Jul 2;20(4):633–40. doi: 10.5811/westjem.2019.4.40031 (PMC6625680; doi:10.5811/westjem.2019.4.40031)
Supplement: Supplementary file 1 [file wjem-20-633-s001.docx]

**Supplement Data 1. Checklist of specific chief complaints during screening.**

- Pain
- Respiratory complaints (dyspnea, cough)
- Stroke like symptoms
- Swollen leg or arm
- Diarrhea
- Dysuria
- Altered mental status (confusion, intoxication, seizure)
- Bleeding
- Syncope
- Psychiatric symptoms (suicidal ideation, psychotic symptoms)
- Skin lesion/reaction
- Fever
- Palpitation
- Nausea with vomiting
- Trauma
